# Insights from a century of data reveal global trends in ex situ living plant collections

**DOI:** 10.1038/s41559-024-02633-z

**Published:** 2025-01-21

**Authors:** Ángela Cano, Jake Powell, Anthony S. Aiello, Heidi Lie Andersen, Thomas Arbour, Aleisha Balzer, Dennise Stefan Bauer, Jeremy Bugarchich, Fernando Cano, Maria Paula Contreras, Robert Cubey, Ignacio Czajkowski, Milton H. Diaz-Toribio, Thomas Freeth, Nicolas Freyre, Martin F. Gardner, M. Patrick Griffith, A. Lovisa S. Gustafsson, Mats Havström, Leslie R. Hockley, Peter M. Hollingsworth, Tina Jørgensen, Kristen Kindl, Donovan Kirkwood, Denis Larpin, Øystein Lofthus, Cornelia Löhne, Adriana López-Villalobos, Dan Luscombe, Dermot Molloy, Clara Morales-Rozo, Inese Nāburga, Anna Nebot, Christoph Neinhuis, Cindy S. Newlander, Joke Ossaer, Greg Payton, Jon Peter, Raul Puente Martinez, Anne-Cathrine Scheen, David Scherberich, Anna Maria Senekal, Clare Shearman, John Siemon, Stephanie A. Socher, Rebecca Sucher, Alex Summers, Joanna M. Tucker Lima, Alison Vry, Jessica Wong, Damian Wrigley, Frédéric You, Samuel F. Brockington

**Affiliations:** 1 Cambridge University Botanic Garden, Cambridge, UK; 2 Longwood Gardens, Kennett Square, PA USA; 3 Department of Natural History, University of Bergen, Bergen, Norway; 4 Holden Forests & Gardens, Kirtland, OH USA; 5 Botanic Gardens of Sydney, Sydney, New South Wales Australia; 6 Botanical Garden, University of Muenster, Muenster, Germany; 7 San Diego Botanic Garden, Encinitas, CA USA; 8 Jardín Botánico Carlos Thays, Buenos Aires, Argentina; 9 Jardín Botánico de Cartagena Guillermo Piñeres, Turbaco, Colombia; 10 Royal Botanic Garden Edinburgh, Edinburgh, UK; 11 Jardín Botánico Francisco Javier Clavijero, Instituto de Ecología, Xalapa, Mexico; 12 Royal Botanic Gardens, Kew, UK; 13 Conservatoire et Jardin Botaniques de la Ville de Genève, Geneva, Switzerland; 14 Montgomery Botanical Center, Miami, FL USA; 15 Rogaland Arboret, Sandnes, Norway; 16 Gothenburg Botanical Garden, Gothenburg, Sweden; 17 Toronto Botanical Garden, Toronto, Ontario Canada; 18 Natural History Museum and Botanic Garden, Copenhagen, Denmark; 19 Desert Botanical Garden, Phoenix, AZ USA; 20 Stellenbosch University Botanical Garden, Stellenbosch, South Africa; 21 Muséum National d’Histoire Naturelle, Paris, France; 22 Botanical Garden, University of Oslo, Oslo, Norway; 23 Botanic Gardens, University of Bonn, Bonn, Germany; 24 University of British Columbia Botanical Garden, Vancouver, British Colombia Canada; 25 The National Pinetum, Bedgebury, Goudhurst, UK; 26 Royal Botanic Gardens Victoria, Melbourne, Victoria Australia; 27 Jardín Botánico José Celestino Mutis, Bogota, Colombia; 28 Botanic Garden of University of Latvia, Riga, Latvia; 29 Botanical Garden of the University of Valencia, Valencia, Spain; 30 Faculty for Biology and Botanic Garden, Technische Universität Dresden, Dresden, Germany; 31 Denver Botanic Gardens, Denver, CO USA; 32 Arboretum Wespelaar, Haacht, Belgium; 33 The Dawes Arboretum, Newark, NJ USA; 34 Royal Botanical Gardens, Hamilton and Burlington, Ontario Canada; 35 Stavanger Botanic Garden, Stavanger, Norway; 36 Jardin Botanique de Lyon, Lyon, France; 37 Wellington Gardens, Wellington, New Zealand; 38 Botanical Garden, Department of Environment and Biodiversity, Paris Lodron University of Salzburg, Salzburg, Austria; 39 Missouri Botanical Garden, Saint Louis, MO USA; 40 National Botanic Garden of Wales, Llanarthney, UK; 41 Westonbirt, The National Arboretum, Tetbury, UK; 42 Chicago Botanic Garden, Glencoe, IL USA; 43 Jardin Botanique Alpin La Jaÿsinia, Samoëns, France; 44 Department of Plant Sciences, Cambridge University, Cambridge, UK

**Keywords:** Plant sciences, Environmental sciences

## Abstract

Ex situ living plant collections play a crucial role in providing nature-based solutions to twenty-first century global challenges. However, the complex dynamics of these artificial ecosystems are poorly quantified and understood, affecting biodiversity storage, conservation and utilization. To evaluate the management of ex situ plant diversity, we analysed a century of data comprising 2.2 million records, from a meta-collection currently holding ~500,000 accessions and 41% of global ex situ species diversity. Our study provides critical insights into the historical evolution, current state and future trajectory of global living collections. We reveal sigmoidal growth of a meta-collection that has reached capacity in both total accessions and total diversity, and identify intrinsic constraints on biodiversity management, including a median survival probability of 15 years. We explore the impact of external constraints and quantify the influence of the Convention on Biological Diversity, which we link to reduced acquisition of wild-origin and internationally sourced material by 44% and 38%, respectively. We further define the impact of these constraints on ex situ conservation but highlight targeted initiatives that successfully mitigate these challenges. Ultimately, our study underscores the urgent need for strategic prioritization and the re-evaluation of ex situ biodiversity management to achieve both scientific and conservation goals.

## Abstract (web summary)

The authors collate a meta-collection of ex situ living plant diversity held in 50 botanical collections worldwide, spanning a century of data and currently containing ~500,000 accessions. Their analyses examine the capacities and constraints within living plant collections, reveal the impact of the Convention on Biological Diversity and its consequences for material exchange and conservation, and call for the re-evaluation of strategic priorities.

## Main

The global network of living collections collectively holds at least 105,634 species, representing 30% of the diversity of all land plant species^1^. These collections already serve a multitude of specialist roles, integrating recreational, educational, scientific and conservation functions^2^. But the emerging challenges of the twenty-first century require a re-evaluation of the roles of living collections, especially for science and conservation. Historically valuable to traditional botanical disciplines, living collections are experiencing a renaissance. Recent research agendas, including biomimetics^3^ and omic-scale technologies^4–6^, are driving renewed demand for diverse and taxonomically accurate living plant collections. And beyond scientific demands, living collections have also been proposed as a vital ex situ repository for plant species threatened with extinction^7^ and valuable source of plants for species and ecological restoration^8^. With >20% of the world’s plant diversity at risk of extinction^9^, living collections are viewed as essential in achieving the former Global Strategy for Plant Conservation^10^ (and target 4 of the Kunming-Montreal Global Biodiversity Framework). Consequently, it is vital that the global network of living collections is collectively managed to meet these goals.

Unlike other botanical collections such as herbaria and seedbanks, living collections are inherently more transient, presenting a unique set of challenges. These collections are often globally sampled, with diverse species cultivated outside their native environments, surviving only through resource-intensive horticulture. Continual loss of plants is counterbalanced by propagating from existing collections or by annually importing new batches of plants, known as accessions, which maintain the collection’s size and diversity. The ongoing balance between plant loss and acquisition underscores the intricate and resource-heavy nature of sustaining living botanical collections. The dynamic turnover also creates a fundamental challenge for managing and using these collections for science and conservation^11^. Maintaining highly diverse assemblages within limited space necessitates a substantial data operation, with every accession monitored and tracked throughout its lifespan, resulting in extensive data accumulation. These data are crucial for effective management and have the potential to offer profound insights into the dynamics of living collections over time.

But despite the extensive accumulation of data, there is a notable lack of comprehensive longitudinal accession-level analysis to understand the constraints affecting living collections, to evaluate their management and to measure progress towards strategic objectives. To address this gap, we have developed an analytical pipeline and applied it to a globally sampled dataset of 50 ex situ living collections (Supplementary Table 1). These 50 collections, termed a meta-collection (sensu ref. ^12^), constitute a substantial subsample, holding ~500,000 accessions, and 41% of the minimum species diversity previously estimated to be held across the global botanic garden network^1^. Using our pipeline, we have parsed ~2.2 million data records covering 100 years (1921–2021). We analyse the dynamics in collection size and diversity metrics, reveal changing patterns in provenance, and infer the influence of international biodiversity legislation. Our analyses illuminate the constraints impacting ex situ conservation, highlight the distinctive characteristics of successful ex situ conservation programmes, and quantify parameters linked to the sustainability of living collections.

## Results

### The meta-collection has reached peak capacity

**Fig. 1:**
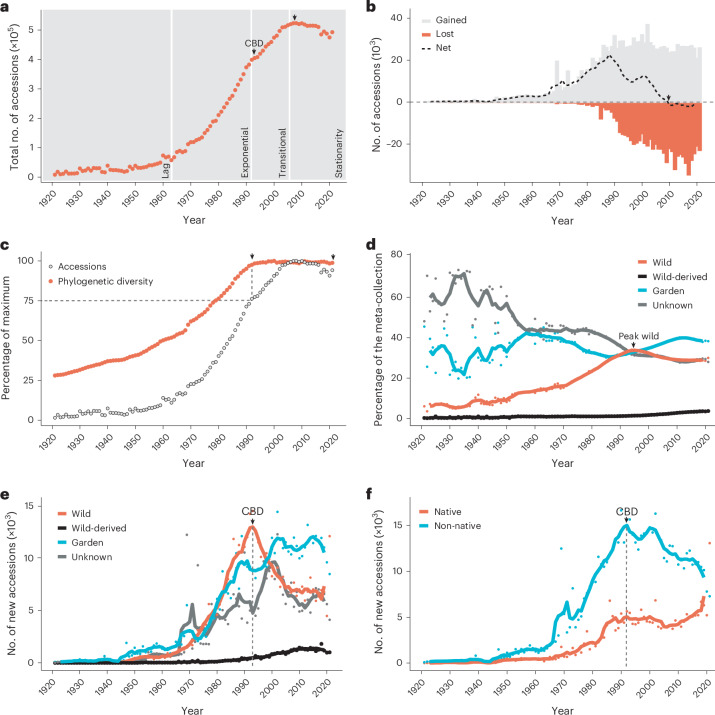
Dynamics of the meta-collection with respect to capacity, diversity and provenance. **a**, Total number of accessions within the meta-collection over time (1921–2021) (black arrow denotes peak capacity reached in 2008; CBD marks the Convention on Biological Diversity coming into force in 1993). **b**, Turnover over time, with dotted line representing the net turnover as a 5 year rolling average (black arrow denotes first occurrence of net negative in 2009). **c**, A comparison of sigmoidal growth curves for accrual of accessions versus accrual of phylogenetic diversity (PD) (black arrows indicate no notable increase in PD over the past 30 years despite 25% increase in collection size). **d**, Changing proportions in provenance as a percentage of total accessions over time, with the line equating to a 5 year rolling mean (black arrow denotes point where proportion of wild accessions had reached its peak—peak wild—in 1995). **e**, Changing proportions in provenance among newly accessioned material, with the lines equating to a 5 year rolling mean (black arrow marks the CBD). **f**, Changing proportion of new accessions of native origin versus non-native origin, with the lines equating to a 5 year rolling mean (black arrow marks the CBD).

To characterize the growth dynamics of living collections over time, we analysed all collections individually (Supplementary Fig. 1) but also combined all 50 living collections and reconstructed the contents of this meta-collection over a 100 year period (1921–2021). We found that growth in number of accessions adheres to a sigmoidal growth curve with distinct lag, accelerated, transitional and stationary phases (Fig. 1a). The meta-collection experienced its most rapid expansion between 1975 and 1992. Following this period, the pace moderated, peaking in 2008, then levelling off and entering a phase of gradual decline from 2015 through 2021. We explored the dynamics underlying stationarity by examining turnover. Here, we observed a lag between the increasing rates of accession gain and the increasing rates of accession loss (Fig. 1b). However, as the rate of incoming accessions drops off, the rate of accession loss continues, leading to a net loss of accessions for the first time in 2009 (Fig. 1b), defining the peak size of the meta-collection. In effect, this sigmoidal pattern signifies density-dependent growth of collections, where the rate of increase is influenced by the existing number of accessions, eventually reaching a saturation point where no further growth can occur due to limited resources. In other words, there is now zero to negative growth, as the meta-collection has reached its carrying capacity, an ecological concept that is commonly applied in the context of sigmoidal density-dependent growth curves^13^.

### The meta-collection has reached peak diversity

**Extended Data Fig. 1:**
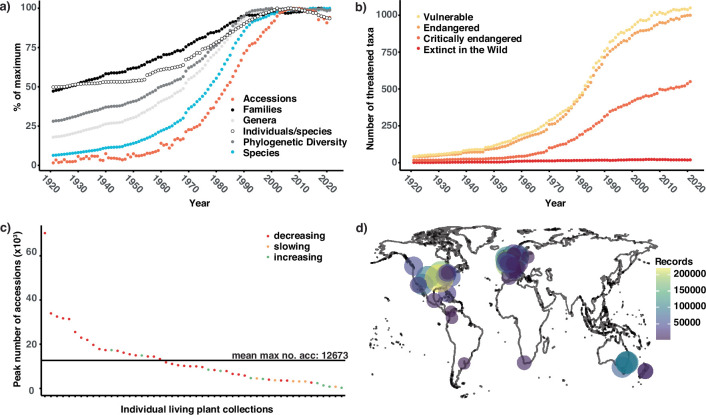
Taxonomic diversity, extinction risk, and species genetic pool across the meta-collection, and its spread in historic capacity and geographic location. **a)** Comparison of change over time in the proportional numbers of accessions, families, genera, and species, as well as phylogenetic diversity in the meta-collection; **b)** Change over time in the number of threatened species in the meta-collection by IUCN Red List categories; **c)** Historic maximum number of accessions in the fifty individual living plant collections. The different colours mirror the arrows in Supplementary Fig. 1 (red being collections decreasing in number of accessions, orange plateauing collections, and green growing collections); **d)** Geographic location of the fifty individual living plant collections that compose the meta-collection, where the diameter of the circles is proportional to their total amount of records.

To understand how the diversity of the meta-collection has changed over time we analysed the accumulation rates at different taxonomic hierarchies. Within the meta-collection, all levels exhibited similar sigmoidal curves, with species, genera and family diversity all reaching stationarity (Extended Data Fig. 1a). We further quantified diversity by calculating changes in phylogenetic diversity (PD) and show that PD had similarly plateaued within the meta-collection (Fig. 1c). Notably, diversity measures reach stationarity earlier than total accessions. For instance, while the overall accession-level capacity of the meta-collection did not peak until 2007, PD had already levelled off by 1990, at a point when the meta-collection was only at 75% of its maximum size. Consequently, expanding the meta-collection from 75% to its maximum size, had a negligible impact on the overall capture of PD (Fig. 1c), with implications for efficiencies in the storage of diversity. Notably the mean number of individuals per species, a common proxy for intraspecific diversity, also reaches stationarity, consistent with the peak of carrying capacity (Extended Data Fig. 1a). That diversity metrics peak ahead of total accessions is in part due to nested taxonomic hierarchies, but other limiting factors may include taxonomic duplication within and among collections, constraints of horticultural expertise limiting the diversity that can be grown, and the temperate ecological niche biases operating across a predominantly global north network of botanic gardens^1^.

### The meta-collection has reached peak wild

We examined provenance, as a critical factor influencing the utility of living collections. We grouped provenance into four categories: wild-origin (sourced directly from nature), wild-derived (documented asexual or sexual propagation from a wild-origin accession), garden-origin (from cultivated collections without a traceable wild link) and unknown-origin. We then analysed how the proportions of these provenance categories changed over time (Fig. 1d). Results show a notable improvement in provenance identification, with unknown origins dropping from a rolling average of 60% to a still high level of 29% in 2021. Wild-origin accessions meanwhile increased from a rolling average of 6% to a current value of 29% in 2021 but peaked in 1995 at 34.1% (termed peak wild) (Fig. 1d). We explored the causes of peak wild, and by examining the provenance of annual introductions to the meta-collection, we observed that wild collections (but not other provenances) showed a remarkable immediate and pronounced downward trend post-1993 (Fig. 1e) ultimately resulting in a 44% reduction in the acquisition of wild-origin material. This decline is most easily attributable to the Convention on Biological Diversity (CBD), which came into force on 29 December 1993, and assigns sovereignty over biodiversity to national governments, creating potential and realized obstacles to the sharing of genetic materials. Given the inferred influence of CBD on wild collections, we then examined whether living collections were also accessioning less internationally sourced material and indeed found a congruent 38% decline in the accessioning of non-native plants post-1993, with a notable mirror increase in accessions of native plants (Fig. 1f). In effect, we find that the proportional and absolute decline in wild and international acquisition are off-set by recycling garden-origin and wild-derived accessions, and by greater accessioning of native species.

### Numerous constraints limit ex situ conservation

**Fig. 2:**
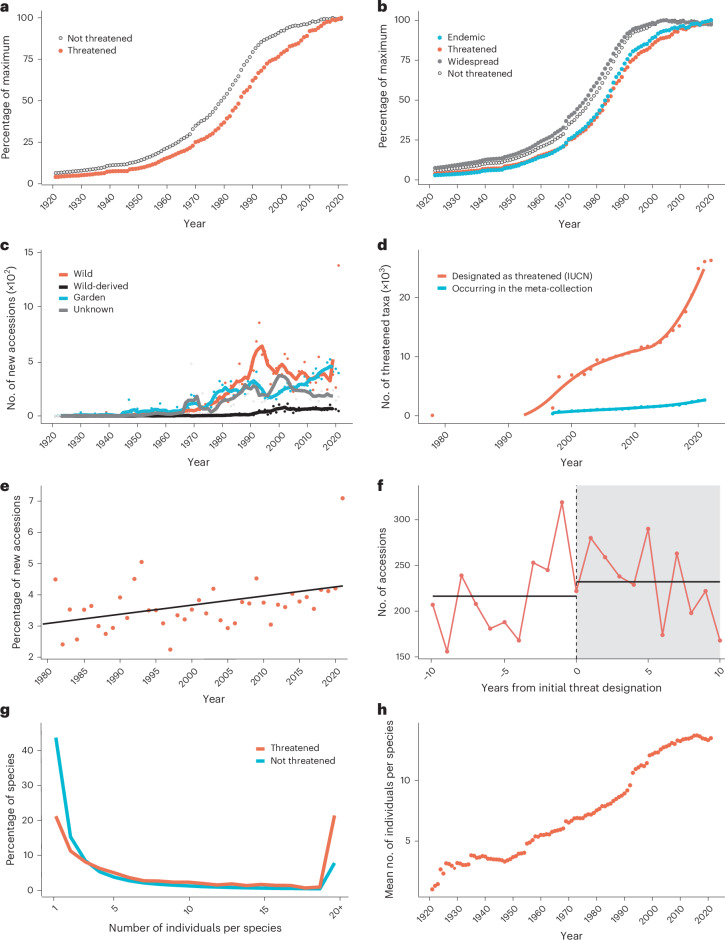
The dynamics of threatened plant collections. **a**, Proportion of threatened versus non-threatened species within the meta-collection over time. **b**, Same as **a** with endemic versus widespread species overlayed for comparison. **c**, Number of new accessions of threatened species by provenance with lines equating to a 5 year rolling mean. **d**, Accumulation of species designated as threatened by the IUCN versus the accumulation of designated threatened species in the meta-collection (starting from the first appearance of digitally available IUCN Red List in 1978). **e**, Proportion of threatened plants accessioned relative to all new accessions in a given year. **f**, Rates of accessioning of threatened species in 10 years before designation versus 10 years after designation (year 0 is when a species was first designated as threatened). **g**, Number of individual plants per species for threatened versus non-threatened plants. **h**, Mean number of individual plants per threatened species over time.

We were curious whether the patterns observed across the meta-collection also extend to the International Union for Conservation of Nature (IUCN) Red-listed plant species threatened with extinction. To assess this, we retrospectively applied current IUCN (December 2023) designations to historical data and examined the growth curves. We found that threatened collections display the same sigmoidal curve as the meta-collection as a whole, indicating that threatened species accumulation is similarly constrained (Fig. 2a). However, threatened collections have not yet fully plateaued and lag behind the trends of the meta-collection as a whole (Fig. 2b and Extended Data Fig. 1b). Interestingly, we saw no visible increase in the rate of accumulation of threatened species with the onset of IUCN designations post-1978 (Fig. 2a). So, we then asked what factors might be constraining the acquisition of threatened species. One obvious constraint is that threatened plants are by definition rare in the wild and, all things being equal, are less likely to be accessioned into living collections. To explore this, we examined the pattern of endemics versus widespread species and found a similar lag pattern for endemics, suggesting that rarity in the wild is a limiting factor for inclusion (Fig. 2b). Importantly, the distinct patterns for threatened species and endemics are not the result of poorer survival rates within collections, as addressed in the subsequent section exploring median survival rates. Using the same dataset with retrospectively applied current IUCN designations we then asked whether the provenance trends observed for the meta-collections as a whole, also applied to the threatened species pool. Here, we found similar trends to that seen for the meta-collections as whole; that is, a clear decline in wild accessions post-1993, and an increasing proportion of threatened plant accessions being recycled as garden-origin or wild-derived post-1993, potentially limiting the accrual rate of new threatened species (Fig. 2c). Given little to no evidence that extra effort is being taken to accrue threatened species at the level of the whole meta-collection, we then sought to understand responsiveness of the meta-collection to threat designation. Here, it is necessary to distinguish between the intentional acquisition of threatened species versus the species that are already in the collections and simply acquiring newly designated threat status. We first compared the rate of threat designation against the rate of addition of threated plants to the meta-collection, noting that extinction risk is accruing at a much faster rate than inclusion in the meta-collection (Fig. 2d). Limited acquisition of threatened species is therefore not due to a limiting pool of threatened species. To exclude the effect of already accessioned species accruing new threat designations, we first examined the relative proportion of threatened to non-threatened species that were newly accessioned each year (1978–2021) and found a very limited increase in the proportion of threatened accessions being newly incorporated (effectively a 1% increase over 40 years) (Fig. 2e). We then investigated whether the number of species added to the meta-collection significantly increased in the 10 years after threat designation (mean = 232.1, s.d. = 42.3) versus 10 years before threat designation (mean = 216.4, s.d. = 48.8), but found no significant effect (*P* = 0.45). Together these two analyses confirm that a lack of response to threat designation contributes to slow accrual rates (Fig. 2f). Finally, we examined the relationship between numbers of species and numbers of individuals per species as this trade-off may constrain the accrual of threatened species. We compared the number of individual plants accessioned for threatened versus non-threatened species, as a proxy for genetic diversity, and found that threatened species tend to have more individuals than non-threatened species (Fig. 2g), consistent with conservation value of intraspecific genetic diversity. However, it is evident that the capacity constraints on the meta-collection as a whole are also impacting on the conservation of intraspecific diversity as the mean number of individuals per threatened species has also reached stationarity (Fig. 2h).

### Effective ex situ conservation leaves measurable signature

**Fig. 3:**
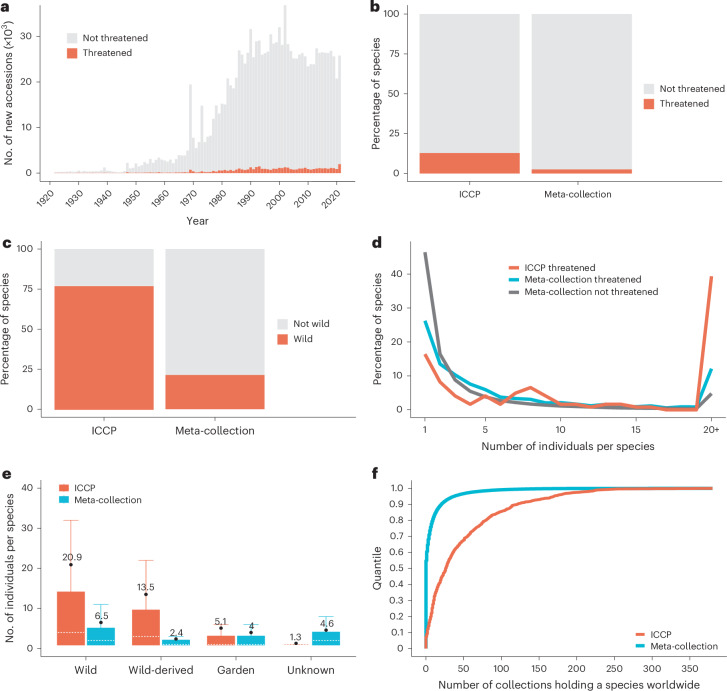
The quantifiable signature of effective ex situ conservation using the International Conifer Conservation Programme as a case study. **a**, Number of new accessions each year in the meta-collection grouped stacked into threatened and non-threatened species. **b**–**f**, Comparison of existing plants in the meta-collection against existing plants in the International Conifer Conservation Programme (ICCP) by showing: the percentage of threatened species (**b**); the proportion of wild-origin taxa (**c**); the number of individual plants per threatened species in the ICCP and in the meta-collection versus non-threatened species in the meta-collection (**d**); the number of individual plants per species by provenance, with box plots depicting median (dotted line) and mean (filled circle with number), upper and lower quartile, whiskers, outliers not included (**e**); and sample quantiles of the number of global collections that threatened species are held in for the ICCP versus the meta-collection (**f**).

At the level of the entire meta-collection, accessioning of threatened species constitutes a small portion of total acquisition effort and this has not changed very much (Fig. 3a). However, it is vital to recognize that certain ex situ conservation programmes can exhibit very different behaviours, such as the International Conifer Conservation Programme (ICCP) which is run out of the Royal Botanic Garden Edinburgh. Here, we compared the ICCP against the meta-collection, across a range of parameters. First, we examined the proportion of threatened species within the ICCP versus the meta-collection as a whole and found a fivefold increase in the proportion of threatened species within the ICCP (Fig. 3b). Then we asked whether the ICCP exhibited an unusual signature with respect to provenance and found a 3.5-fold increase in wild collected species within the ICCP versus the meta-collection (Fig. 3c). We explored the extent to which the ICCP capture more genetic diversity by using the number of individual plants per species as a proxy and found that a higher proportion of species were represented by a larger number of accessioned individuals (Fig. 3d). Moreover, the extra resource allocated to capturing more individuals per species is much more apparent for conservation-relevant categories wild-origin and wild-derived, than for garden- or unknown-origin accessions (Fig. 3e). The ICCP seeks to intentionally distribute threatened species across its distributed network of safe sites, as an insurance policy and to empirically evaluate optimum ex situ localities for long-term survival. We therefore asked whether the ICCP threatened species were more globally distributed and so less rare in cultivation, relative to species in the meta-collection as a whole, and found that this was indeed the case (Fig. 3f).

### Median survival in the meta-collection is largely immutable

**Fig. 4:**
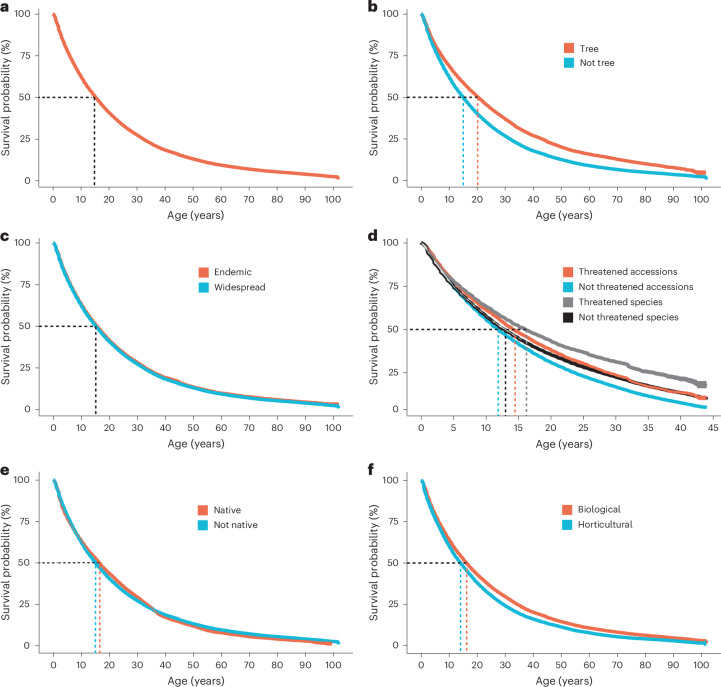
Kaplan–Meier survival curves as applied to the meta-collection. The survival curves are shown as solid lines, with 95% confidence intervals (CI) (shaded regions); because of the size of the datasets and the high degree of confidence, the shaded CIs are often barely visible. The median survival probability (MSP) for each group is indicated by the dashed lines. **a**–**f**, Estimated MSP of: all accessions across the meta-collection, MSP = 15 yr (*n* = 1,018,769, CI = 15.3–15.4) (**a**); trees and non-trees, MSP = 20 and 15 yr, respectively (trees—*n* = 69,616, CI = 20.1–20.7; non-trees—*n* = 949,153, CI = 15.0–15.1) (**b**); endemic and widespread species, MSP = 15 yr for both (endemic—*n* = 153,107, CI = 15.6–15.9; widespread—*n* = 865,662, CI = 15.2–15.3) (**c**); threatened accessions and species restricted to plants that were accessioned between 1980 and 2021 (**d**) (the MSP times for threatened versus non-threatened accessions was 14 versus 12 years (threatened accessions—*n* = 35,664, CI = 13.9–14.3; non-threatened accessions—*n* = 840,750, CI = 11.8-11.9) and threatened versus non-threatened species was 16 versus 13 years (threatened species—*n* = 13647, CI = 15.7–16.6; non-threatened species—*n* = 447,561, CI = 12.7–12.9)); native and non-native species, MSP = 16 and 15 yr, respectively (native—*n* = 137,150, CI = 16.5–16.6; non-native—*n* = 944,351, CI = 15.0–15.1) (**e**); biological (species, subspecies, varieties and forma) and horticultural (cultivars and hybrids) taxa MSP = 16 and 14 yr, respectively (biological—*n* = 704,704, CI = 16.2–16.4; horticultural—*n* = 253771, CI = 14.2–14.4) (**f**).

Given the challenging constraints identified in our previous analyses, we then leveraged our longitudinal dataset to understand the persistence of accessions in the meta-collection using Kaplan–Meier survival curves. Here, we adopt a broad definition of survival, to include natural loss as well as deliberate de-accessioning, which together contribute to the transience of living collections. Across the entire meta-collection, we found median survival probability to be 15 years (Fig. 4a), which increased to 20 years for tree collections alone (Fig. 4b). Further segmentation of the data yielded additional insights. For example, we expected to see poorer median survival rates for endemic species due to their more constrained native niche but actually found no difference in median survival between endemic versus non-endemic species, both at 15 years (Fig. 4c). We then evaluated threatened accessions post-onset of digital IUCN Red Lists (1978–2021). Like endemics, threatened species are commonly narrow in native distribution, but in contrast to endemics versus non-endemics, threatened species exhibit a marginally better median survival probability than non-threatened accessions (14 versus 12 years), indicating a small if positive signal of targeted ex situ conservation activity (Fig. 4d). Given that the ex situ conservation of a species over time is contingent on the collective accessions for a given species we further examined the persistence of threatened versus non-threatened species rather than accessions and likewise observed similar improvement in median survival probability for threatened species (16 versus 13 years). Remarkably, we observed the same median survival probability for native and non-native accessions (Fig. 4e), and, contrary to received horticultural wisdom, horticultural cultivars have lower median survival probability (13 years) compared to biological species (16 years), potentially due to selective inbreeding of sport traits unrelated to survival (Fig. 4f). In the context of living collections management, data recording for ‘birth’ (incoming accessions) tends to be more accurate than data recording for ‘death’ (outgoing accessions), which can lag, and so median survival probability values are likely to be upper estimates. But all in all, although certain subcategories of collections such as trees and threatened plants shift the dial to a degree, a median survival probability of ~15 years seems largely immutable across different subsets.

## Discussion

In this study we have analysed the first globally sampled accession-level longitudinal dataset for an ex situ living plant meta-collection. The emergence of sigmoidal growth curves, characteristic of biological systems constrained by limited resources, is unsurprising yet revealing. Our findings indicate that the meta-collection has reached its carrying capacity^13^ in both the volume of accessions (Fig.1a) and botanical diversity (Fig. 1c and Extended Data Fig. 1a). The data comprising our meta-collection were challenging to acquire, as the global network of living collections has a notable global north bias and constitutes a fragmented data ecosystem with limited adoption of data standards and a closed data culture, with many datasets not passing the requirements of this study. Despite this, we globally sampled a variety of institutions across a continuous size range (Extended Data Fig. 1c,d), to capture patterns and processes representative of the global network. Individual collections experience unique trajectories influenced by their specific circumstances, histories and management practices. Most collections within our meta-dataset are slowing in rate, plateauing or decreasing (40/50). Some generally smaller collections are continuing to expand (10/50); however, this is counterbalanced by substantial decreases in more extensive collections (Extended Data Fig. 1c). It is highly likely that the patterns that we report extend to the network as a whole given the propensity of collections in the global north^1^, which have mostly plateaued or are decreasing in size. Newer collections are being established, including in the global south, but our meta-collection analysis suggests that the limited size and number of these collections cannot currently compensate for deceleration in the older, larger and more numerous collections in the global north. Collectively therefore, the overarching trends point towards capacity constraints, underscoring a critical need for strategic prioritization. It is important to emphasize that decreasing size is not in itself a negative, and, at the level of individual collections, can be the signature of better collection management. Nonetheless, if the global network has indeed reached peak capacity, peak diversity and peak wild, as our data imply, then we will need to find ways to fulfil emerging scientific opportunities and deliver solutions to the global biodiversity crisis, with existing capacity and within the existing constraints.

Provenance is a critical property that can influence downstream utility. Wild-origin collections are the most valuable for a range of applications including conservation, species and ecosystem restoration, as well as most lines of scientific enquiry. Among the more striking insights from our research are the dynamic shifts in provenance, apparently influenced by the CBD, which assigns sovereignty over biodiversity to national governments and entered into force in 1993. Immediately following CBD coming into force we see a marked decrease in wild collections, culminating in a 44% reduction in the acquisition of wild-origin specimens (Fig. 1d). The concomitant increases in the recycling of wild-derived and garden-origin material (Figs. 1d and 2c) probably reflects an attempt to safeguard key plants within the meta-collection as well as restrictions to sourcing material directly from the wild. A second distinctive trend we observe is decline in the international exchange of material post-CBD, culminating in a 38% reduction, which is lately mirrored by increases in the accessioning of native material (Fig. 1e). Collectively these shifts suggest growing regionalization which could fundamentally reshape the future composition of botanical collections and the partitioning of diversity across the global network. The potential or realized influence of CBD has been subject to considerable debate, but the discussion to date has primarily been anecdotal and anticipatory rather than quantitative and evidential^14–19^. The dramatic shifts in provenance that we have linked to CBD constitute compelling quantitative evidence for the global impact of CBD on genetic exchange and are testament to the influence of this widely ratified biodiversity legislation. The strength and clarity of the signal is due to the transience of living collections, which requires a substantial annual influx of genetic material and is therefore sensitive in reporting constraints that affect that influx. Given this sensitivity, it is interesting that the Nagoya protocol^20^, which came into force in 2014 and legally enforces aspects of the CBD, appears to have had minimal immediate impact on the overall meta-collection (but note Fig. 1f), in contrast to the CBD effect. One interpretation might be that the acquisition culture within the meta-collection is already in alignment with CBD after 1993 and is therefore less sensitive to the Nagoya protocol than might have been expected. But it is early days, and time will tell. We do, however, observe considerable numbers of recently accessioned specimens of unknown provenance (Fig. 1e), highlighting ongoing challenges in achieving traceability^16^ with implications for adherence to the Nagoya protocol and the availability of appropriate material for species and ecological restoration. Accessioning of wild-origin material remains depressed relative to pre-1993 highs, with significant implications for the conservation value and scientific utility of collections and signalling a need for renewed collaborative collecting efforts under the appropriate legal and ethical frameworks.

The patterns observed for threatened species within living collections reveal challenges and opportunities. On the one hand, the number of threatened plant species held within the meta-collection is increasing, the proportion of threatened to non-threatened accessions incorporated on an annual basis shows a modest increase of 1% (over 40 years), there is marginally improved survival of threatened species (+3 years) versus non-threatened species within the meta-collection and greater capture of individuals (a proxy for genetic diversity) for threatened versus non-threatened plants (Figs. 2a,e,g and 4d). But, on the other hand, rate of accessioning of threatened plants is in transition to stationarity, inclusion of globally threatened plants has been negatively affected by biodiversity legislation, the rate at which plants are being designated as threatened substantially exceeds their rate of inclusion in the meta-collection, there is little evidence of responsiveness to threat designation within a 10 year window, and carrying capacity is limiting the number of accessioned individuals per threatened species (Fig. 2a–d,f,h). Given the collections-wide impact of CBD, ensuring that ex situ conservation practices and related research can proceed apace in the face of international legislation is a key challenge^19^. The generalized median survival probability of 15 years across the entire meta-collection (close to an earlier estimate of 13 years based on a single collection^11^) provides a key baseline against which living collections must contend, if ex situ conservation is to be effective in the long-term. All the more so, as ex situ conservation will need to factor in both population genetics and survival rates, that will require orders of magnitude more sampling than is currently achieved for most threatened species^21^, in the context of a carrying capacity that is limiting intraspecies sampling (Fig. 2h). More broadly, given the lack of compelling signal at the level of the entire meta-collection there is clearly a challenge in shifting the culture of living collections to act not only as repositories of threatened diversity, but as active participants in the conservation of species. In this regard, the ICCP exemplifies a measurably effective conservation approach. The ICCP collections hold proportionally more threatened species, are wilder in provenance, capture more individuals per species, with threatened species distributed to a greater extent across the global network (Fig. 3).

In conclusion, our study reveals the dynamics of globally connected living plant collections, providing critical insights into their historical evolution, current state and future trajectories. Living plant collections remain a crucial resource, but the challenges of managing these collections are profound, given their largely immutable median survival probabilities, inherent resource limitations and external constraints that include international biodiversity legislation. At a minimum, future success will depend on addressing the complexities of living collection management, and adapting to intrinsic and extrinsic constraints, especially in the face of changing environmental and legislative contexts. Data and data analysis will be central to innovation and will require investment in digital infrastructure, data-driven thinking and an open data culture. Finally, given our inference that most living collections in the global north will have reached stationarity, we must think about how to achieve global objectives, by rethinking how we allocate existing resource and by collectively investing in capacity-building and development of connected living collections in the global south. Living collections in the global south tend to be younger and hold less diversity, yet are often in closer proximity to biodiversity hotspots, and may be less limited by some of the geopolitical constraints quantified in this study. Our study therefore underscores the importance of maintaining a collaborative approach among botanic gardens, conservationists and the broader scientific community, to innovate and adapt. Living collections have a rich history of adaptation, in responding to evolving societal demands and in delivering innovation. Despite the challenges we have identified, in an era of climate change and accelerating biodiversity loss, the need for high-quality and resilient living collections to deliver nature-based solutions has never been greater.

## Methods

### Data gathering, standardizing and enrichment

Between 2021 and 2024, we contacted institutions across the global living collections network to solicit participation in the study. Following an initial round of responses, we conducted interviews with ~100 institutions to better understand their data collecting and data storage practices, and to evaluate the potential of their data for inclusion within the study. Subsequent to these interviews we obtained data from 89 institutions, which we manually examined, and then screened for compatible data standards and data completeness. Following this screening, we were left with 50 living plant collections from 19 countries and 5 continents, including Africa (1 collection), Australasia (6 collections), Eurasia (27 collections), North America (13 collections) and South America (3 collections) (Supplementary Table 1). We then standardized and enriched individual datasets by performing taxonomic name and authority matching to Plants of the World Online (POWO), whereby our matching algorithm detected common mistakes in the taxonomic name such as incorrect infraspecific level and simple typos and checked for partial author matches. Once matched, when differences occurred, we performed a taxonomic standardization following the nomenclature of POWO. We then used the standardized taxonomic names and authority to enrich further and obtained the following information: (1) the extinction risk from the IUCN Red List^22^; (2) the number of institutions holding a given taxon from an extract from PlantSearch (by the organization Botanic Garden Conservation International, BGCI) taken from ref. ^1^; and (3) whether a taxon is or is not a tree from GlobalTreeSearch (BGCI; v.1.7). POWO contains geographic information on taxa as Biodiversity Information Standards Taxonomic Databases Working Group (TDWG) geographical codes^23^ expressed to the third level (level 3) of that system, which divides the world into 369 distinct geographic regions, commonly referred to as botanical countries. We enriched each taxon name with a list of the botanical countries they occur in and determined whether a taxon is endemic (occurs in a single country) or widespread (occurs in more than one country). We provide the meaning and standard values of all fields taken forward for analysis in Supplementary Table 2. We combined all 50 collections into a single dataset, termed the meta-collection. We included records of both alive and dead plantings and excluded material other than living plantings (for example, seeds, herbarium and propagation records). We restricted the data window of our analyses to a century from 1921 to 2021, as a sufficient window to capture key patterns. See Supplementary Table 2 and Extended Data Fig. 1 for more details.

### Capacity analyses

We used the meta-collection to construct two types of datasets that underpin most of the analyses in the study: historical reconstructions of the entire meta-collection and turnover datasets. We reconstructed the historic state of the entire collection every year by using a combination of the accession year and most recent status update which gave us a date on whether a plant is alive or dead—this allowed us to determine which plants were existing in each year. To assess growth of the collections, we used the historic reconstructions to then count the number of accessions in the meta-collection each year. We generated turnover datasets by separating out new and lost accessions for each year. We filtered new accessions per year by using the accession year to determine when an accession was gained by a collection. We used the year when the last individual of an accession died to filter lost accessions in any given year. We determined net turnover by calculating the difference between new and lost accessions each year. Using the same methodology as above, we also performed historic reconstructions on individual collections to visualize trends per collection. We then manually inspected the resulting curves and categorized individual collections as increasing, decreasing or plateauing. See also Fig. 1a,b and Supplementary Fig. 1.

### Diversity analyses

Using the historical reconstructions obtained for the meta-collection capacity analyses above, we counted the number of species, genera and families occurring in the meta-collection in each year. As another measure to understand how diversity changed over time, we used PD^24^, which calculates the total distance of the minimum spanning path of branches connecting a set of species in their phylogeny. Herein, we used the seed plant phylogeny created by ref. ^25^. Given that not all species in the meta-collection were included in that phylogeny, we measured the PD at the genus level instead, whereby we trimmed the phylogeny to include one species per genus using tools from the ape R package^26^. This means that, we calculated the PD by determining the total distance of the minimum spanning path of branches connecting the genera existing in the meta-collection each year. PD computation used the Picante R package^27^. See also Fig. 1c and Extended Data Fig. 1.

### Provenance analyses

Using the same historical reconstructions obtained for the capacity analyses, we counted the number of accessions for each provenance type (Supplementary Table 2) occurring each year in the meta-collection. To further understand the changing trends in the sourcing of plants, we used the turnover dataset to count the number of incoming accessions each year by their provenance type. We explored a different concept of provenance, using the notion of native and non-native, where we considered the geographic distribution of plants relative to the location of the collection in which they were contained. Since botanical countries are the building blocks of the geographic distribution of plants, we defined the native region of a living collection as the region described by selecting the minimum number of TDWG level 3 regions that contain the political country of origin of the living collection. For example, a collection located in Argentina would have a native region of the merged botanical countries of Argentina Northeast, Argentina South and Argentina Northwest. We defined a species as native to a given collection if the geographic distribution of the taxon overlapped with that of the native region of the collection. A species was considered non-native if there was no overlap. We then used the turnover dataset, to count the number of native and non-native accessions acquired each year by the meta-collection. See also Fig. 1d–f.

### Threatened species analyses

We defined threatened species as IUCN Red List categories: vulnerable, endangered, critically endangered and extinct in the wild. We defined non-threatened to include, not evaluated, data deficient, least concern and near threatened. We looked at the accumulation of threatened species in the meta-collection in two different ways. First, we retrospectively mapped current Red List designations (1 December 2023) to the historic reconstructions for the entire meta-collection and counted the accumulation of threatened and not-threatened accessions to establish their relative growth curves. To ascertain whether there was any change in behaviour with respect to the acquisition of threatened species due to the onset of IUCN Red listing (1978), we included all years contained in the meta-collection (1921–2021). Second, we used the IUCN Red List assessment history extracted using the rredlist R package^22^ to establish which species are designated as threatened between 1978 and 2021, where threatened species also include the now defunct IUCN categories: extinct/endangered and rare, and we used the assessment date rather than the publication date. We then compared the accumulation of designated threatened species, with their incorporation into the meta-collection. We applied local regression^28^ to find trends in growth for both the threatened species incorporated into the meta-collection and the number of Red List threatened species as both appear to grow nonlinearly over time. To assess responsiveness to threat designation, we only considered species that were first assessed to be threatened between 2000 and 2009, where the species remains threatened afterwards. This provided 788 taxa whose accessions in the meta-collection we analysed. For each taxon we found the number of accessions from 10 years before the assessment year to 10 years afterwards. We then combined the accessions across all taxa by using the ‘net’ year from individual assessments and tested for significant differences using a Welch two-sample *t*-test (two-sided; *t* = −0.77; 95% CI −58.66–27.26; effect size Cohen’s *d* = −0.34; d.f. = 17.65; *P* = 0.4521). To investigate trends in accessioning of threatened species we used the turnover dataset to extract the proportion of all newly acquired accessions that were classed as threatened each year since 1978, and we used linear regression to illustrate the change over time. We also, filtered the above data into provenance categories and calculated the proportion of wild-origin, wild-derived, garden-origin and unknown-origin new threatened accessions each year. To assess whether threatened status affects duplication within collections, we counted the number of individuals accessioned since 1978 across threatened and not-threatened species and then grouped species by their total number of individuals. Since the distribution of the number of individuals per species was positively skewed, we grouped together species that had 20 or more individuals to improve data visualization. We converted the number of species to the proportion of species by dividing the counts by the total number of species. See also Figs. 2 and 3.

### ICCP analyses

To detect the signature of effective ex situ conservation programmes we individually enriched and analysed the dataset of the ICCP and compared a series of parameters of their currently existing plantings against those existing in the meta-collection as a whole. In particular, we compared the proportion of threatened (sample sizes: ICCP = 123 and meta-collection = 2,646) against non-threatened taxa (sample sizes: ICCP = 668 and meta-collection = 103,377), the proportion of taxa of wild-origin (sample sizes: ICCP = 612 and meta-collection = 22,732) against other-origins (sample sizes: ICCP = 179 and meta-collection = 83,243), the number of individual plants for each threatened and non-threatened species and the number of individual plants per taxon for the different provenance categories (sample sizes: garden ICCP = 143 and meta-collection = 118,409; unknown ICCP = 25 and meta-collection = 66,111; wild ICCP = 647 and meta-collection = 69,706; wild-derived ICCP = 79 and meta-collection = 12,142). We also analysed the extent to which plants were shared across the global network of living collections by extracting sample quantiles across the number of global collections each taxon was held in for the meta-collection and ICCP (after removing indeterminate taxa). See also Fig. 3b–f.

### Median survival

To investigate the effects of biological and horticultural properties on the lifespan of accessions in botanic gardens, we used survival analysis^29^ which is a branch of statistics for analysing the expected duration of time until an event, in our case, the loss of an accession. We used this approach because it uses the duration of plants in living collections that are still present, defined as right-censored observations in survival analysis. To perform the analysis, we required the duration—time from accessioning to either accession death or the last status update—and whether the last status update corresponded to a death for each accession. We extracted the duration of each accession by calculating the difference between the most recent status date and the accessioning date. The ‘existing status’ at the most recent status update date was used to determine whether accessions were alive or dead. We used the Kaplan–Meier estimator^30^ to perform survival analysis and generated Kaplan–Meier curves. We determined the median survival from Kaplan–Meier curves by taking the expected time for half the plants to have died. For the survival of species in living collections, we manipulated the accession survival information, before applying the survival analysis outlined for accessions. For each species in a living collection, we found all accessions corresponding to the species, extracted date intervals where at least one accession was always alive, and converted the date intervals into time durations. For each time duration, we determined whether the species was dead or alive at the end of the interval. If a species had more than one survival window (that is, the species was reintroduced into a living collection), then each survival window was treated as a separate survival time in the analysis. See also Fig. 4.

### Reporting summary

Further information on research design is available in the Nature Portfolio Reporting Summary linked to this article.

## Supplementary information

Supplementary Information Supplementary Fig. 1 and Tables 1 and 2.

Reporting Summary

## Data Availability

The living collections data that support the findings of this study are available via GitHub at https://github.com/cubg-curation/DESLPC/releases/tag/v1.0.0; to ensure the safety of the specimens, the location of individual accessions has been anonymized. The following data sources used in the enrichment of botanic garden datasets are publicly available online: BGCI GlobalTreeSearch (v.1.7, https://tools.bgci.org/global_tree_search_trees_1_7.csv) and World Checklist of Vascular Plants (v.11, https://sftp.kew.org/pub/data-repositories/WCVP/Archive/). IUCN Red List was obtained using the rredlist R package (v.0.7.1).

## Code availability

All analyses were performed on R (v.4.2.3) where, more specifically, the following packages were used: tidyverse (v.2.0.0), pbapply (v.1.7-2), stringr (v.1.5.1), ggplot2 (v.3.5.1), scales (v.1.3.0), zoo (v.1.8-12), picante (v.1.8.2), readxl (v.1.4.2), dplyr (v.1.1.4), reshape (v.0.8.9), ggpubr (v.0.6.0), utils (v.4.2.3), ivs (v.0.2.0), ggsurvfit (v.1.0.0), survival (v.3.5-3) and openxlsx (v.4.2.5). IUCN Red List information was obtained using the rredlist package^22^, phylogeny tree manipulation and PD computation used the ape^26^ and picante^27^ packages, respectively, and survival analysis was performed using the survival package^30^. World Checklist of Vascular Plants (v.11) was downloaded from POWO. Custom code was used to enrich garden records with WCVP, BGCI GlobalTreeSearch, BGCI PlantSearch and IUCN Red List information. LivingCollectionDynamics R package is available via GitHub at https://github.com/cubg-curation/LivingCollectionDynamics.
