# Supplementary material for: Insights from a century of data reveal global trends in ex situ living plant collections
Source: Nat Ecol Evol. 2025 Jan 21;9(2):214–24. doi: 10.1038/s41559-024-02633-z (PMC11807835; doi:10.1038/s41559-024-02633-z)
Supplement: Supplementary file 1 — Supplementary Fig. 1 and Tables 1 and 2. [file 41559_2024_2633_MOESM1_ESM.pdf]

nature ecology & evolution

Supplementary information

https://doi.org/10.1038/s41559-024-02633-z

Insights from a century of data reveal global trends in ex situ living plant collections

In the format provided by the authors and unedited

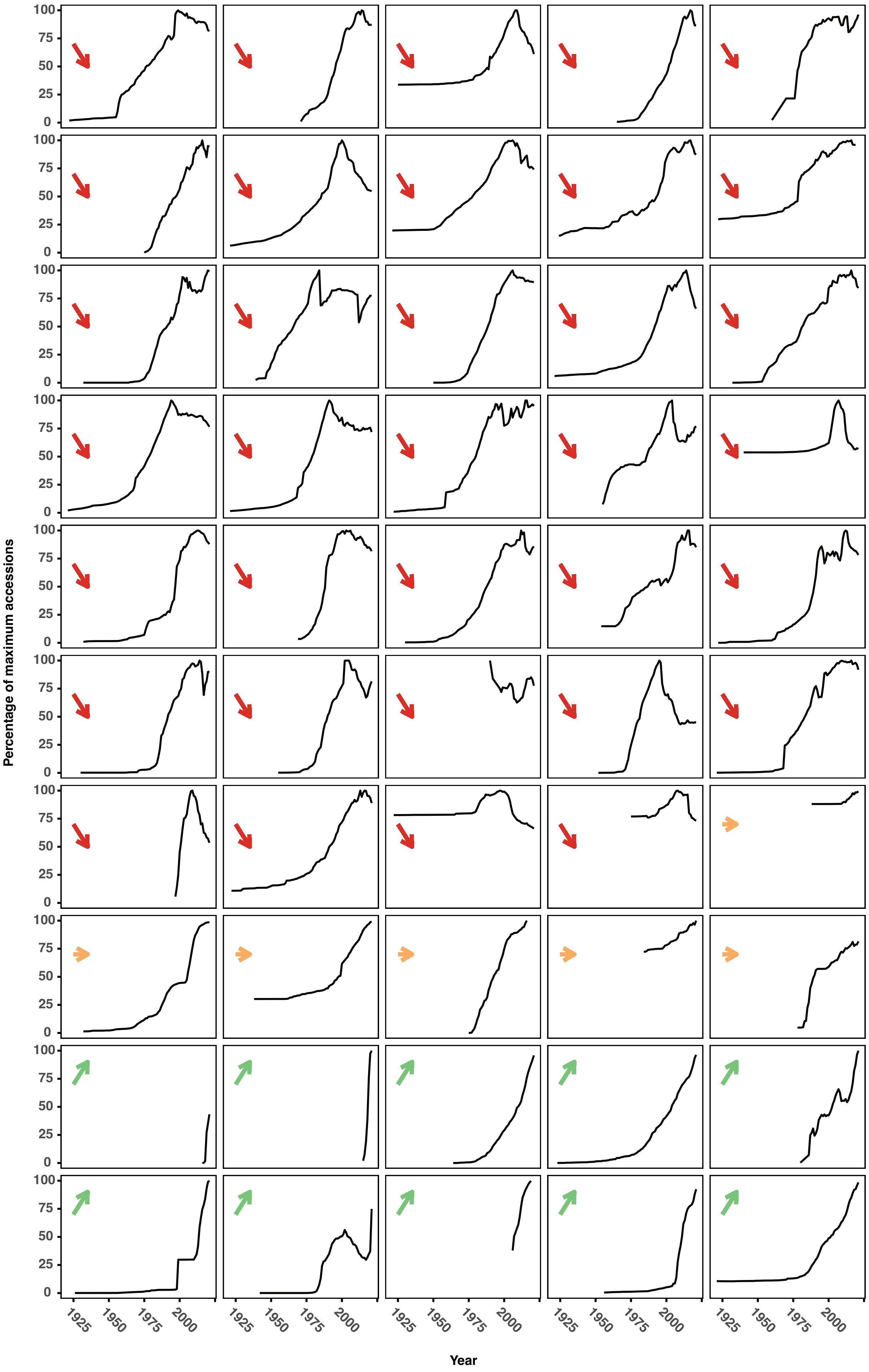

Supplementary Fig. 1. Change in the number of accessions over time (1921-2021) in fifty individual living plant collections. The absolute number of accessions was transformed to percentage of maximum to allow for comparison across collections. Arrows represent the current trend of a given collection, with red being collections that are decreasing in number of accessions, orange are plateauing or transitioning collections, and green are growing collections.

Table 1. List of the living collections included in this study, including name, country in which each collection is located, and the year of their first digital accession.

Country	Living plant collection	Year of 1st accession
Argentina	Jardín Botánico Carlos Thays	1898
Australia	Australian Botanic Garden Mount Annan	1900
Australia	Blue Mountains Botanic Garden Mount Tomah	1871
Australia	Royal Botanic Garden Sydney	1828
Australia	Victoria Royal Botanic Garden	1962
Austria	Botanischer Garten Salzburg	1984
Belgium	Wespelaar Arboretum	1900
Canada	British Columbia University Botanic Garden	1952
Canada	Royal Botanical Gardens Burlington	1930
Canada	Toronto Botanic Garden	2006
Colombia	Jardín Botánico de Bogotá	2016
Colombia	Jardín Botánico de Cartagena	2015
Denmark	Botanical Garden, Natural History Museum of Denmark, University of Copenhagen	1796
France	Jardin botanique alpin de la Jaÿsinia	1906
France	Jardin Botanique de Lyon	1850
France	Jardin botanique Val Rahmeh - Menton	1902
Germany	Botanische Garten der Technischen Universität Dresden	1910
Germany	Botanische Gärten der Universität Bonn	1750
Germany	Münster Botanic Garden	1840
Latvia	Latvia University Botanic Garden	1900
Mexico	Jardín Botánico Francisco Javier Clavijero Botanic Garden	1884
New Zealand	Ōtari-Wilton's Bush	1922
New Zealand	Wellington Botanic Garden	1840
Norway	Bergen University Gardens	1900
Norway	Oslo University Botanic Garden	1900
Norway	Rogaland Arboret	1975
Norway	Stavanger botaniske hage	1978
South Africa	Stellenbosch University Botanical Garden	1926
Spain	Universitat de Valencia Jardí Botanic	1990
Sweden	Göteborgs botaniska trädgård	1900
Switzerland	Conservatoire et Jardin botaniques de la Ville de Genève	1800
UK	Bedgebury National Pinetum and Forest	1975
UK	Benmore Botanic Garden	1820
UK	Cambridge University Botanic Garden	1846
UK	Dawyck Botanic Garden	1800
UK	Kew Royal Botanic Garden	1867
UK	Logan Botanic Garden	1902
UK	Royal Botanic Garden Edinburgh	1892
UK	Wakehurst	1879
UK	National Botanic Garden, Wales	1997
UK	Westonbirt, The National Arboretum	1829
USA	Chicago Botanic Garden	1965
USA	Denver Botanic Garden	1932
USA	Desert Botanic Garden	1939
USA	Holden Arboretum and Cleveland Botanic Garden	1932
USA	Longwood Gardens	1955
USA	Missouri Botanical Garden	1939
USA	Montgomery Botanical Center	1932
USA	San Diego Botanic Garden	1880
USA	The Dawes Arboretum	1918

Table 2. Living plant collections database fields required for our analyses, including definition and examples of their contents.

Name of the field	Description
**Accession number**	**Accessions** are plant material acquired by the collection as one or more individual plants (items) and identified by a unique accession number, which is a series of digits that usually begins by the accessioning year, followed by a serial number. Used in our analyses to count the number of accessions E.g. 20211234
**Accession number + Item qualifier**	Accession number with qualifier that identifies the **items** (individual plants) of an accession. Used in our analyses to count the number of items. E.g. 20211234*A, 20211234*B
**Accession year**	Year of accession. E.g. 2021
**Conservation status**	As defined by the IUCN Red List. E.g. Not evaluated, Data deficient, Least Concern, Near Threatened, Vulnerable, Endangered, Critically endangered, Extinct in the wild.
**Conservation status binary**	Based on the above field, but considering taxa in the categories of Vulnerable, Endangered, Critically endangered, and Extinct in the wild taxa as threatened, and all other categories as Not Threatened.
**Distribution**	Categorical distribution of a taxon to a TDWG geographical code expressed to that system's third level.
**Family**	Plant family where the taxon is classified. E.g. Arecaceae
**Genus**	Plant genus where the taxon is classified. E.g. *Areca*
**Genus-species**	Species binomial name excluding authority and any infraspecific or horticultural precision. Used in our analyses to count the number of species. E.g. *Areca catechu*
**Item status date**	Date when an item was last updated. E.g. 25/06/2021
**Item status type**	Status of an item. E.g. Existing = alive, NotExisting = dead
**Item type**	Type of item. E.g. Planting, Seed, Propagation
**Number of Collections**	Number of ex-situ collections holding a taxon according to a PlantSearch extract[1]
**Provenance**	Provenance type of an accession E.g. wild-origin (sourced directly from nature), wild-derived (discernibly descended from a wild-origin accession), garden-origin (from cultivated collections without a traceable wild link), and unknown-origin
**Taxon name**	Taxonomic name of an accession, including infraspecific (e.g. subsp., var., f.) and horticultural (hybrids, cultivars) categories, but excluding authority name. Used in our analyses to count the number of taxa. E.g. *Areca catechu* var. *alba*, *Areca catechu* 'Princess Elizabeth'
**Taxon name + authority**	Same as above but including authority name. When available, we use this field for taxonomic standardisation; otherwise, we use Taxon name. E.g. *Areca catechu* L.
**Taxonomic level**	species, subspecies, variety, forma, hybrid, cultivar, or indeterminate
**Tree**	Indicates whether the taxon is a tree or not, obtained by comparing taxonomic names to those in GlobalTreeSearch
