# Supplementary material for: Insights from a century of data reveal global trends in ex situ living plant collections
Source: Nat Ecol Evol. 2025 Jan 21;9(2):214–24. doi: 10.1038/s41559-024-02633-z (PMC11807835; doi:10.1038/s41559-024-02633-z)
Supplement: Supplementary file 2 — Reporting Summary [file 41559_2024_2633_MOESM2_ESM.pdf]

nature portfolio

Corresponding author(s): Brockington, SF

Last updated by author(s): Jul 8th 2024

Reporting Summary

Nature Portfolio wishes to improve the reproducibility of the work that we publish. This form provides structure for consistency and transparency in reporting. For further information on Nature Portfolio policies, see our Editorial Policies and the Editorial Policy Checklist.

Statistics

For all statistical analyses, confirm that the following items are present in the figure legend, table legend, main text, or Methods section.

n/a	Confirmed	
☐	☒	The exact sample size (*n*) for each experimental group/condition, given as a discrete number and unit of measurement
☐	☒	A statement on whether measurements were taken from distinct samples or whether the same sample was measured repeatedly
☐	☒	The statistical test(s) used AND whether they are one- or two-sided<br>*Only common tests should be described solely by name; describe more complex techniques in the Methods section.*
☒	☐	A description of all covariates tested
☒	☐	A description of any assumptions or corrections, such as tests of normality and adjustment for multiple comparisons
☐	☒	A full description of the statistical parameters including central tendency (e.g. means) or other basic estimates (e.g. regression coefficient) AND variation (e.g. standard deviation) or associated estimates of uncertainty (e.g. confidence intervals)
☐	☒	For null hypothesis testing, the test statistic (e.g. *F*, *t*, *r*) with confidence intervals, effect sizes, degrees of freedom and *P* value noted<br>*Give P values as exact values whenever suitable.*
☒	☐	For Bayesian analysis, information on the choice of priors and Markov chain Monte Carlo settings
☒	☐	For hierarchical and complex designs, identification of the appropriate level for tests and full reporting of outcomes
☐	☒	Estimates of effect sizes (e.g. Cohen's *d*, Pearson's *r*), indicating how they were calculated

Our web collection on statistics for biologists contains articles on many of the points above.

Software and code

Policy information about availability of computer code

Data collection	Red List information was obtained using the rredlist R package (version 0.7.1).<br>BGCI PlantSearch: No software used.<br>BGCI GlobalTreeSearch: No Software used.<br>WCVP: No Software used.
Data analysis	All analyses were performed on R (version 4.2.3) where more specifically the following packages were used tidyverse(2.0.0), pbapply(1.7-2), stringr(1.5.1), ggplot2(3.5.1), scales(1.3.0), zoo(1.8-12), picante(1.8.2), readxl(1.4.2), dplyr(1.1.4), reshape(0.8.9), ggpubr(0.6.0), utils(4.2.3), ivs(0.2.0), ggsurvfit(1.0.0), survival(3.5-3) and openxlsx(4.2.5). IUCN RedList information was obtained using the rredlist package22, phylogeny tree manipulation and Phylogenetic Diversity computation used the ape26 and picante27 packages, respectively, and survival analysis was performed using the survival package30. World Checklist of Vascular Plants (Version 11) was downloaded from Plants of the World Online. Custom code was used to enrich garden records with WCVP, BGCI's GlobalTreeSearch, BGCI PlantSearch and IUCN Red List information.<br>LivingCollectionDynamics R package: https://github.com/cubg-curation/DESLPC

For manuscripts utilizing custom algorithms or software that are central to the research but not yet described in published literature, software must be made available to editors and reviewers. We strongly encourage code deposition in a community repository (e.g. GitHub). See the Nature Portfolio guidelines for submitting code & software for further information.

Data

Policy information about availability of data

All manuscripts must include a data availability statement. This statement should provide the following information, where applicable:

- Accession codes, unique identifiers, or web links for publicly available datasets
- A description of any restrictions on data availability
- For clinical datasets or third party data, please ensure that the statement adheres to our policy

The living collections data that support the findings of this study are available on Github (https://github.com/cubg-curation/DESLPC/releases/tag/v1.0.0), to ensure the safety of the specimens, the location of individual accessions has been anonymised. The following data sources used in the enrichment of botanic garden datasets are publicly available online: BGCI GlobalTreeSearch (Version 1.7, https://tools.bgci.org/global_tree_search_trees_1_7.csv) and World Checklist of Vascular Plants (Version 11, https://sftp.kew.org/pub/data-repositories/WCVP/Archive/). IUCN Red List was obtained using the rredlist R package (version 0.7.1).

Research involving human participants, their data, or biological material

Policy information about studies with human participants or human data. See also policy information about sex, gender (identity/presentation), and sexual orientation and race, ethnicity and racism.

Reporting on sex and gender	N/A
Reporting on race, ethnicity, or other socially relevant groupings	N/A
Population characteristics	N/A
Recruitment	N/A
Ethics oversight	N/A

Note that full information on the approval of the study protocol must also be provided in the manuscript.

Field-specific reporting

Please select the one below that is the best fit for your research. If you are not sure, read the appropriate sections before making your selection.

☐ Life sciences ☐ Behavioural & social sciences ☒ Ecological, evolutionary & environmental sciences

For a reference copy of the document with all sections, see nature.com/documents/nr-reporting-summary-flat.pdf

Ecological, evolutionary & environmental sciences study design

All studies must disclose on these points even when the disclosure is negative.

Study description	We analyzed a globally sampled dataset comprising fifty ex-situ living collections, collectively termed a meta-collection. Our analyses sought to describe the trends and trajectories inherent within the meta-collection, and to report on the hitherto undescribed and unquantified dynamics of ex-situ plant biodiversity management. This meta-collection includes approximately 500,000 accessions, representing 41% of the species diversity previously estimated across the global botanic garden network. The dataset encompasses ~1.9 million records spanning a century (1921-2021). In addition to information associated with the records as received, we enriched the data with information on whether records pertained to trees, species threatened with extinction, and the rarity of species in cultivation. Our analyses generally covered the entire 100-year period, reconstructing the meta-collection's composition by considering plant acquisition and loss dates. Additionally, we examined various subsets of these data based on attributes such as provenance (wild, wild-derived, garden, or unknown origin), threatened versus non-threatened species, and trees versus non-trees. Sometimes we sub-sampled across a narrower time window, for example with respect to threatened species, as designation of extinction risk did not begin until the 1970s. The sizes of these sub-samples are detailed in the manuscript.
Research sample	The research sample comprised living collections data from 50 different living collection sampled globally, collectively comprising some 2.2 million records.
Sampling strategy	Statistical methods were not used to determine sampling size. The data comprising our meta-collection were challenging to acquire, as the global network of living collections has a Global North bias and constitutes a fragmented data ecosystem with limited adoption of data standards and a closed data culture, with many data sets not passing the requirements of this study. So we simply used all data we were able to obtain. But we sought to sample a range of institutions of different sizes, and spanning different geographic regions and cultures, as much as possible. The subsample we have obtained is small relative to total number of collections but large in terms of the diversity it holds, with some 42% of all species diversity held across the global botanic garden network.

Data collection	Between 2021 and 2024, we contacted institutions across the global living collections network in order to solicit participation in the study. Following an initial round of responses, we conducted interviews with ~100 institutions in order to better understand their data collecting and data storage practices, and to evaluate the potential of their data for inclusion within the study. Subsequent to these interviews we obtained data from 89 institutions, which we manually examined, and then screened for compatible data standards and data completeness. Following this screening, we were left with data from 50 living plant collections.
Timing and spatial scale	Data was obtained between 2021 and 2024, the data used within the study spans a time period of 100 years. The 50 living plant collections that were sampled are located in 19 countries and 5 continents, including Africa (1 collection), Australasia (6 collections), Eurasia (27 collections), North America (13 collections), and South America (3 collections).
Data exclusions	We focussed on a timeline of 100 years, because living collection data records deteriorate with age, and we found that 100 years was adequate to report all of the interesting patterns that emerged from the data. This window was in effect empirically determined.
Reproducibility	All analyses were repeated numerous times to confirm reproducibility, and we have not reported any analyses that failed the test of reproducibility
Randomization	Data was allocated into groups according to how it had been denoted in the living collections databases. The experiments were not pre-designed, and were based on previously collected data, and so we were unable to account for any co-variates in our analyses.
Blinding	Individual living collections were assigned a random number so identity was limited during analysis. Identity of individual collections was not ascertainable in the context of the meta collections when all data was merged, and the focus of the study was the meta-collection.

Did the study involve field work? ☐ Yes ☒ No

Reporting for specific materials, systems and methods

We require information from authors about some types of materials, experimental systems and methods used in many studies. Here, indicate whether each material, system or method listed is relevant to your study. If you are not sure if a list item applies to your research, read the appropriate section before selecting a response.

Materials & experimental systems

n/a	Involved in the study	
☒	☐	Antibodies
☒	☐	Eukaryotic cell lines
☒	☐	Palaeontology and archaeology
☒	☐	Animals and other organisms
☒	☐	Clinical data
☒	☐	Dual use research of concern
☒	☐	Plants

Methods

n/a	Involved in the study	
☒	☐	ChIP-seq
☒	☐	Flow cytometry
☒	☐	MRI-based neuroimaging

Plants

Seed stocks	N/A
Novel plant genotypes	N/A
Authentication	N/A
